# Comment on “Adjacent segment infection after surgical treatment of spondylodiscitis” by Siam AE et al.

**DOI:** 10.1007/s10195-016-0401-3

**Published:** 2016-03-14

**Authors:** Alberto Di Martino

**Affiliations:** Department of Orthopaedics and Trauma Surgery, University Campus Bio-Medico of Rome, Rome, Italy

Spinal infections are an uncommon but significant clinical scenario that often requires aggressive medical therapy, sometimes leading to surgery [1]. Surgery is indicated in the presence of pathological fractures, neurological deficits, epidural abscess formation, septicaemia resistant to targeted antibiotic therapy, intractable pain, and unacceptable sagittal or coronal plane deformity. Surgery in patients affected by spondylodiscitis includes neurological decompression to preserve or restore the neurological function and to control pain; it is usually performed by laminectomy and/or removal of the infected tissue, and it is usually associated with stabilization of the spine for restoration and preservation of the spinal alignment by the use of titanium-based instrumentation [2].

Siam et al. [3] report on a retrospective cohort of 23 patients operated on for spinal infections who after decompression and fusion surgery developed a spondylodiscitis at the adjacent disc; for such condition these patients required a revision surgery, which consisted of extensive debridement, attempt at fusion, and extension of the instrumentation to the adjacent levels. However, these patients showed high morbidity and mortality rates, with eventual severe neurological compromise.

The authors stress the role of the superinfection of the local haematoma after surgery, and are convinced that the haematogenous route represents the main mechanism of infection of the adjacent disc. However, they also suggest that a direct infection of the adjacent disc space may occur by direct contamination during surgery because of the violation of the disc space by drilling or because of screw malpositioning [4].

No role has been suggested by the authors for the use of titanium-based instrumentation, and in particular for the use of cages in these patients, even though their role in this surgery is still a major issue among surgeons. The use of metal implants in the infected spine has been avoided in the past because of the known adherence of bacteria to metal surfaces. However, experimental studies showed a variable bacterial adherence to different metal surfaces depending on biofilm formation and species. Titanium implants have been used in the setting of spinal infections, because these showed a reduced bacterial biofilm adherence; moreover, similar results and fusion rates were observed when titanium implants or bone grafts were used in surgery for spondylodiscitis [1].

Of the eleven patients with positive microbiological findings described in the study, eight had a recurrence of the same microorganism with multiple antimicrobial drug resistance, and three had a superadded infection with another organism. The high recurrence of the same microorganism poses a question about the influence of the general health status of patients on the genesis of this disease [5]. Moreover, an infection adjacent to a previous operated level may also mean that the primary site of infection (e.g. cardiac valve or urinary tract) was not adequately managed, or that the infection recurred; this renders a comprehensive diagnostic workup mandatory when dealing with such patients.

Twelve out of the 23 patients in the study had no germs retrieved after cultural sample harvest, and the diagnosis of infection was made by clinical and radiological examinations. In orthopaedic surgery, a similar scenario is often observed in the case of suspected joint arthroplasty infections, and general guidelines have been implemented into clinical practice to improve the chances of isolating the responsible microorganisms [6]; however, in spinal surgery this approach is still far from being fully realized.
